# Tumour characterisation, staging and operability assessment in ovarian carcinoma: whole body diffusion-weighted MRI versus CT

**DOI:** 10.1186/1470-7330-15-S1-P40

**Published:** 2015-10-02

**Authors:** K Michielsen, I Vergote, R Vanslembrouck, E Mussen, F Amant, K Leunen, P Moerman, S Fieuws, F De Keyzer, G Souverijns, S Dymarkowski, V Vandecaveye

**Affiliations:** 1 Department of Radiology, Leuven Cancer Institute, University Hospitals Leuven, KU Leuven, Belgium; 2 Department of Obstetrics and Gynaecology, Division of Gynaecologic Oncology, Leuven Cancer Institute, University Hospitals Leuven, KU Leuven, Belgium; 3 Department of Pathology, Leuven Cancer Institute, University Hospitals Leuven, KU Leuven, Belgium; 4 Department of Public Health and Primary Care, Leuven Cancer Institute, University Hospitals Leuven, KU Leuven, Belgium; 5 Department of Radiology, Jessa Hospitals, Hasselt, Belgium

## Aim

To prospectively evaluate whole body diffusion-weighted MRI (WB-DWI/MRI) for tumour characterisation, staging and prediction of complete (R0)-resection compared with computed tomography (CT) in patients with suspected ovarian carcinoma.

## Methods

One-hundred-sixty-six patients suspected for ovarian carcinoma underwent 3T WB-DWI/MRI using 2 b-values (b=0-1000 s/mm^2^), T2-weighted and contrast-enhanced T1-weighted sequences in addition to contrast-enhanced CT. WB-DWI/MRI and CT were independently and blindly evaluated and correlated with pathological findings at surgery as reference standard. Superiority was assessed using two-tailed McNemar tests for following parameters: characterisation of the malignant nature and primary origin of the ovarian mass, assessment of disease extent according to FIGO stage and prediction of R0-resection according to predefined operability criteria. Inter observer agreement for WB-DWI/MRI and CT was determined using Cohen’s kappa statistics.

## Results

For characterisation of malignancy, WB-DWI/MRI showed significantly higher accuracy compared with CT (93 versus 82%, p=0.001). WB-DWI/MRI correctly depicted a non-ovarian malignant mass in 24/32 (75%) of cases compared to only 6/32 (19%) for CT (p<0.001). WB-DWI/MRI assigned more ovarian carcinoma patients to the correct FIGO stage (71/94, 76%) compared with CT (39/94, 41%). For prediction of R0-resection, WB-DWI/MRI showed significantly higher sensitivity (95 versus 80%), specificity (92 versus 74%) and accuracy (94 versus 77%) compared with CT (p=0.039, p=0.012 and p<0.001, respectively). Interobserver agreement was moderate to almost perfect (κ=0.53-1.00) for WB-DWI/MRI and slight to moderate (κ=0.04-0.52) for CT.

## Conclusion

WB-DWI/MRI is superior to CT for lesion characterisation, staging and operability assessment of ovarian cancer justifying its development for pre-operative assessment of ovarian cancer patients.

